# Small extracellular vesicles: crucial mediators for prostate cancer

**DOI:** 10.1186/s12951-025-03326-w

**Published:** 2025-03-21

**Authors:** Sijie Tang, Huiying Cheng, Xueyan Zang, Jiawei Tian, Zhongli Ling, Lingling Wang, Wenrong Xu, Jiajia Jiang

**Affiliations:** 1 The Aoyang Cancer Institute, Affiliated Aoyang Hospital of Jiangsu University, 279 Jingang Blvd, Zhangjiagang, Suzhou, 215600 China; 2 Department of Urology, Affiliated Aoyang Hospital of Jiangsu University, 279 Jingang Blvd, Zhangjiagang, Suzhou, 215600 China; 3 School of Medicine, Jiangsu University, 301 Xuefu Road, Zhenjiang, 212013 China

**Keywords:** sEVs, Heterogeneity, Tumor microenvironment, Prostate cancer

## Abstract

Small extracellular vesicles (sEVs) play a critical role in the progression, diagnosis, and treatment of prostate cancer (PCa), particularly within the tumor microenvironment (TME). Acting as novel biomarkers and agents for targeted biological therapy, sEVs contribute significantly to improving patient survival. These vesicles transport a variety of biomolecules, including proteins, nucleic acids, and lipids, which are instrumental in remodeling the TME, facilitating intercellular communication, and influencing key processes such as tumor growth, metastasis, and therapy resistance. A thorough understanding of sEV heterogeneity, including their biogenesis, characteristics, and potential applications, is essential. Recent advances have illuminated the origins, formation processes, and molecular cargo of PCa-derived sEVs (PCa-sEVs), enhancing our understanding of their role in disease progression. Furthermore, sEVs show promise as diagnostic markers, with potential applications in early detection and prognostic assessment in PCa. Therapeutically, natural and engineered sEVs offer versatile applications, including drug delivery, gene therapy, and immunomodulation, underscoring their potential in PCa management. This review delves into the substantial potential of sEVs in clinical practices for PCa.

**Graphical Abstract:**

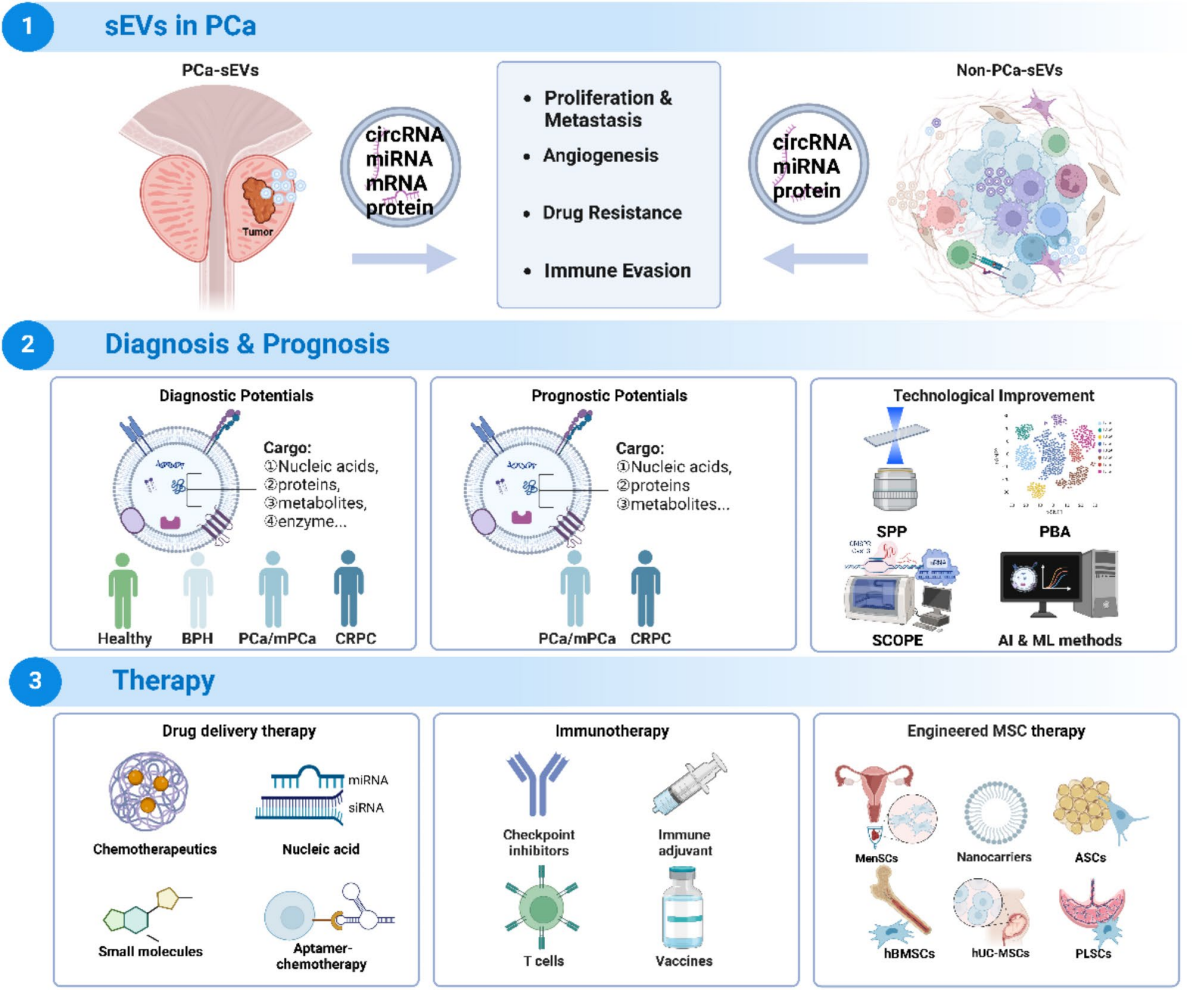

## Introduction

Prostate cancer (PCa), the second most commonly diagnosed cancer in men globally [1], poses significant challenges in management due to its high incidence, metastatic potential, and therapeutic resistance. PCa originates from the prostate epithelium and is potentially curable in its early stages. However, it progresses to more aggressive forms, such as castration-resistant prostate cancer (CRPC) and aggressive variant prostate cancer (AVPC), particularly during metastasis. CRPC arises when androgen signaling persists despite androgen-deprivation therapy (ADT). Genetic mutations in tumor suppressor genes, including BRCA1, BRCA2, and AR, along with the dysregulation of non-coding RNAs, contribute to the heterogeneity of the disease and its resistance to chemotherapy. Moreover, chromatin remodeling and epigenetic changes play a pivotal role in driving this progression. Addressing these challenges requires targeted molecular therapies and a deeper understanding of the molecular networks that regulate PCa progression and therapy resistance [2–4]. Additionally, with current research highlighting the pivotal role of small extracellular vesicles (sEVs) in modulating tumor progression, metastasis, and therapy response [5]. sEVs, also known as exosomes, are important for understanding cell-to-cell communication in PCa [6, 7]. These vesicles, derived from various cell types, including tumor cells and non-tumor cells such as fibroblasts, immune cells, endothelial cells, and stromal cells, play a crucial role in the tumor microenvironment (TME) and significantly influence the pathophysiology of PCa. TME is responsible for preserving the structural integrity and stability of the tissue housing the cells, while also overseeing the functional behaviors of these cells [8, 9]. It is associated with the intricate diversity within the innate immune pathway, primarily stemming from cellular heterogeneity and evident through numerous signaling pathways. One notable example is the cGAS-STING pathway, recognized for its role in suppressing tumorigenesis by maintaining cellular homeostasis [10]. sEVs contribute to the remodeling of the TME and the formation of pre-metastatic niches, facilitating communication between cancer cells and the microenvironment, impacting processes like metastasis, immune regulation, and drug resistance [11–13]. PCa patients frequently develop bone metastases, complicating the disease further. Consequently, research is increasingly focused on areas such as bone metastasis and tumor suppression [14]. PCa EVs promote metastasis by facilitating intercellular communication with bone marrow myeloid cells, activating NF-κB signaling, and enhancing osteoclast differentiation [6]. Treatment with Radium-223 increases immune checkpoint expression in sEVs released from the adverse osteosclerotic metastatic PCa (mPCa) bone microenvironment. Changes in bone TME induced by Radium-223 can be identified through RNA profiling of plasma sEVs [15]. 

sEVs exhibit heterogeneity in their size, shape, cargo, and function, which is critical for modulating cellular interactions within the TME [16]. As molecular couriers, sEVs transport nucleic acids, proteins, and lipids, contributing to signaling processes in the TME, influence cellular interactions, as well as PCa progression and metastasis [17]. Different populations of PCa sEVs can carry distinct sets of proteins that influence the behavior of recipient cells. These sEVs facilitate the horizontal transfer of oncogenic factors between cancer cells and are being investigated as potential biomarkers for PCa [5, 18, 19]. Specific biomarkers, such as microRNAs (miRNAs) or proteins within sEVs, provide valuable insights into tumor characteristics, including aggressiveness, resistance to therapy, and prognosis [20]. Post-translational modifications (PTMs) on proteins, such as acetylation, glycosylation, palmitoylation, and SUMOylation, play crucial roles in the biogenesis, function, and stability of sEVs. For instance, the epigenetic regulation of acetylation inhibits sEV-PD-L1 secretion, significantly enhancing the efficacy of PD-L1 blockade therapy. Cavin-1 expression alters glycosylation modifications on the surface of EVs derived PCa cell line PC3, consequently impacting their internalization [21]. These modifications not only influence the sorting of proteins into sEVs but also modulate intercellular communication, thereby influencing PCa progression and metastasis.

In addition to diagnostic surveillance and prognostic evaluation, sEVs present therapeutic potential in PCa by improving the efficiency of drug delivery. For instance, paclitaxel-loaded sEVs have shown promise, as well as delivering therapeutic RNAs like siRNAs to silence oncogenes such as SIRT6 and Survivin [22–24]. Intriguingly, mesenchymal stem cell (MSC)-derived sEVs have demonstrated potential in enhancing anti-tumor effects. For example, MSC-derived sEVs inhibit the proliferation, migration, and invasion of PC3 cells, while also promoting apoptosis [25]. The engineered sEVs has introduced new possibilities for PCa therapy. Human MSC (hMSC) transfected with siRNA have been shown to significantly inhibit the proliferation of PC3 cells via exocytosis/endocytosis/exosome pathways [26]. Despite the therapeutic potential of hMSC-sEVs, the precise mechanisms within the PCa TME are not yet fully elucidated. For the optimal use of sEVs in cancer treatment, these therapeutic agents must either be delivered in close proximity to the cancer site or possess the ability to target these locations, which also presents certain obstacles. Moreover, there is no consensus on the best approaches for isolating and quantifying sEVs, leading to inconsistent research outcomes and impeding reproducibility [27]. This lack of standardization presents a challenge to translating laboratory findings into effective clinical applications. This aim of this review is to investigate the heterogeneity of sEVs in PCa, emphasizing their diagnostic and prognostic potential. In contrast to existing literature, this article delves deeper into how sEVs remodel TME homeostasis and their functional roles in PCa progression, therapy resistance, and metastasis, while highlighting emerging strategies for targeted drug delivery, gene therapy, and immunomodulation.

## Characterization of sEVs

sEVs, which are a distinct subset of extracellular vesicles (EVs), typically have diameters ranging from 30 to 150 nm [28, 29]. Different from larger vesicles like microvesicles and apoptotic bodies, sEVs play a crucial role in cell-to-cell communication by acting as carriers of molecular information. sEVs have been identified and isolated from diverse body fluids such as blood, urine, and saliva. Their inherent stability makes sEVs appealing for therapeutic and diagnostic applications [30, 31]. Different types of EVs originate from the cell’s endosomal system or being directly shed from the plasma membrane. Distinguished from the traditional sEV generation pathway, in PCa, caveolin 1 (CAV1) is encapsulated through the formation and maturation of autophagosomes and is subsequently released into the extracellular space via the fusion of autophagosomes with the plasma membrane [32]. Protein marker analysis has shown that specific compounds, such as sitafloxacin, forskolin, SB218795, fenoterol, nitrefazole, and pentetrazol, can inhibit the production and release of sEVs in PCa cells [33]. Interestingly, membrane phase separation is essential in sEV biogenesis, facilitating endocytosis through organized membrane domains. Palmitoylation of membrane proteins is instrumental in this process [34]. Furthermore, this phase separation guarantees the inclusion specific molecules, such as miRNA, which are recruited by YBX1 phase-separated droplets [35]. Besides sEVs, ectosomes and supermeres also belong to the category of nano-sized vesicles and particles, playing significant roles in intercellular communication and biological research [36]. A study exploring clinically relevant cargo of extracellular nanoparticles like sEVs and exomeres analyzed the proteomic and RNA composition of the human colorectal cancer cell line, DiFi. Surprisingly, they discovered supermeres, which have unique morphological characteristics compared to sEVs and show significantly higher in vivo uptake, indicating potential as circulating biomarkers and therapeutic targets for various diseases [37]. 

sEVs exhibit remarkable heterogeneity due to their diverse cellular origins, including epithelial cells, fibroblasts, immune cells and stromal cells [38–40]. Each cell type bestows a unique molecular signature onto the sEVs, which functions as crucial mediators of intercellular communication, influencing tumor cell activities and modulating the TME. For instance, stromal cells enhance the radioresistance of PCa cells by secreting IL-8-containing sEVs in the TME [41]. Tumor-associated macrophages (TAM) mediated PCa progression is partially linked to the aberrant expression of miR-95 in TAM-derived sEVs [42]. Studies have isolated sEVs from various PCa cell types, including osteoblastic, osteoclastic, and mixed PCa cell lines, revealing the enrichment of specific RNA in sEVs [43]. Urinary sEVs isolated from patients revealed the enrichment of specific RNA, such as PCA3 and TMPRSS2-ERG [44]. In PCa, sEVs carrying molecules like prostate-specific antigen (PSA) provide diagnostic insights [45]. Recent studies have employed proteomic and cholesterol liquid chromatography-mass spectrometry (LC-MS) analyses to examine sEVs from the serum and urine of healthy individuals and those with castration-resistant PCa (CRPC). They identified consistently present six sEV proteins in both types of biological fluids for CRPC [46]. sEVs contain various RNA molecules, such as circRNA and miRNA [47, 48], which are crucial for PCa survival and metastasis. In PCa cases, sEVs also carry fragments of tumor DNA [49]. Lipids like cholesterol and phosphatidylcholine differ between urinary sEVs and those from cell lines, impacting their potential use as PCa biomarkers [50]. sEVs are emerging as significant contributors to PCa across various stages, with diverse roles in modifying the TME and serving as promising candidates for liquid biopsy.

## PCa-derived sEVs

**Fig. 1:**
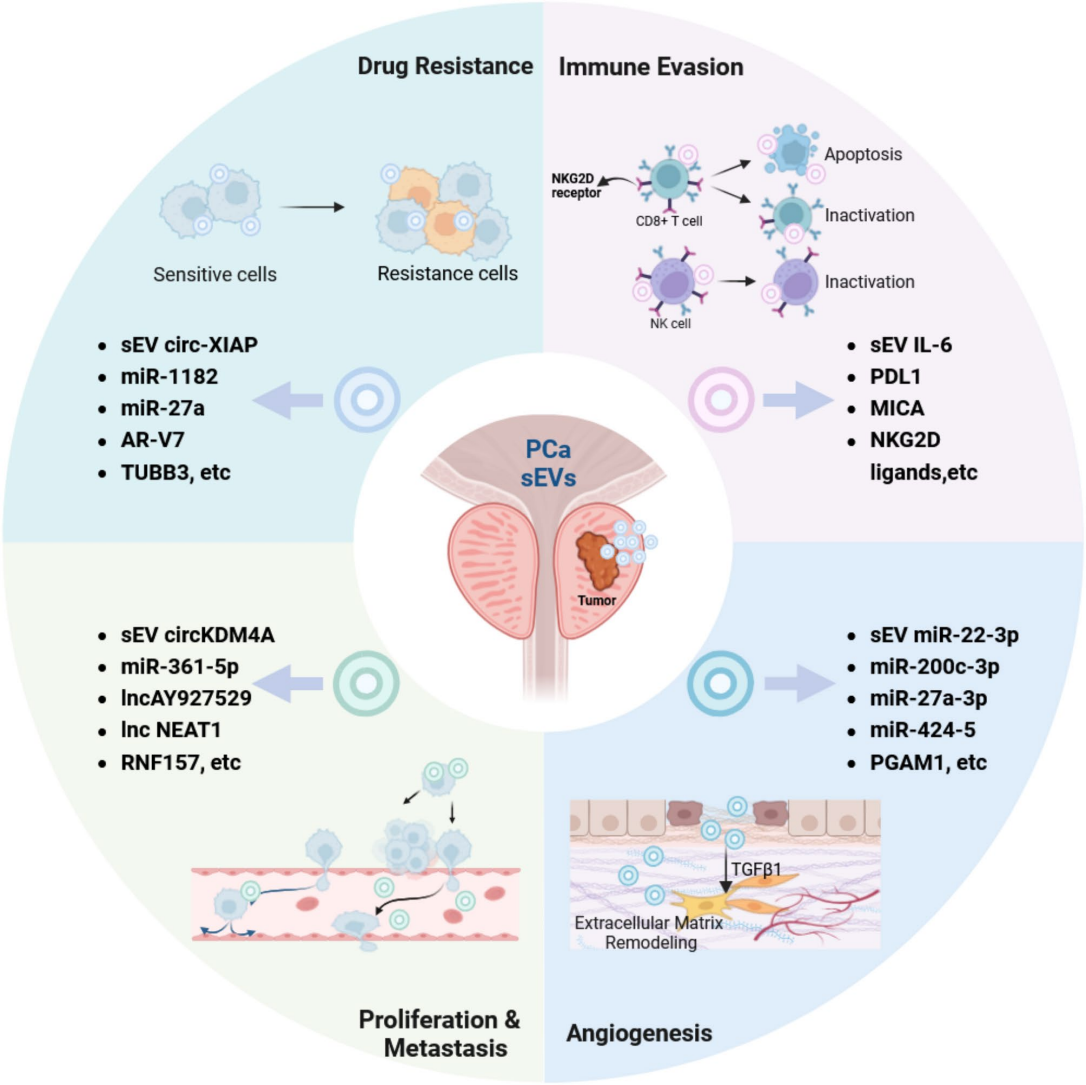
Role and mechanisms of PCa-sEVs in promoting PCa progression. PCa-sEVs promote proliferation, metastasis, angiogenesis, drug resistance, and immune evasion by carrying various molecules, including circRNA, miRNA, lncRNA, and proteins

sEVs exhibit functional diversity in regulating the TME homeostasis. By carrying a range of bioactive molecules, they serve as potent signaling messengers, fostering an environment conducive to PCa growth and survival [51]. Research shows PCa-derived sEVs (PCa-sEVs) interact with stromal cells and remodel the extracellular matrix (ECM), promoting angiogenesis and cancer progression (Fig. 1).

### ECM remodeling and angiogenesis

Within the TME, the stroma, composed of fibroblasts, immune cells, and ECM, interacts significantly with tumor cells, influencing disease progression. The ECM shapes the TME by storing bioactive molecules that drive ECM remodeling [52]. sEVs produced in a stiffened ECM promote tumor growth via Notch signaling activation [53] Activation of a myofibroblast-rich stroma is a critical step in cancer progression. PCa-sEVs induce fibroblast differentiation via TGFβ1, leading to myofibroblast formation, which supports angiogenesis and tumor growth [54]. This suggests a collaborative contribution of sEVs and the ECM to tumor invasion. Moreover, the peri-tumoral collagen network not only serves as a protective barrier but also serves as a dynamic structural scaffold. sEVs also enhance prostate stromal cells (PrSCs) migration through Hyal1 activity, improving adhesion to collagen [55]. The combined actions of ECM degradation and synthesis lead to significant alterations in the mechanical properties of the TME, thereby influencing tumor cell behavior [56]. sEVs transmit specific molecular signals that direct fibroblast differentiation into pro-tumorigenic phenotypes, such as cancer-associated fibroblasts (CAFs). Hypoxic sEVs promote prostasphere formation in both LNCaP and PC3 cells and enhanced α-SMA (a CAF biomarker) expression in PrSCs [57]. Angiogenesis, essential for tumor growth and metastasis, is significantly influenced by sEVs, which either enhance or inhibit new blood vessel formation. sEVs derived from PCa cells like LNCaP and PC3 promote endothelial cell invasiveness and tube formation, with specific miRNAs (e.g., miR-22-3p, miR-27a-3p, and miR-424-5p) playing key roles [58]. Another study highlighted the role of sEV-PGAM1 in facilitating podosome formation and neovascular sprouting in HUVECs [59]. sEVs transfer αvβ6 integrin to human microvascular endothelial cells 1 (HMEC1), promoting angiogenesis in PCa progression [60]. Vascular endothelial growth factor (VEGF) plays a pivotal role in stimulating endothelial cells and driving both normal and pathological angiogenesis [61]. Recent studies also emphasize the influence of sEVs in modulating angiogenic factors like VEGF, thereby accelerating tumor angiogenesis and progression [62, 63]. Intriguingly, PCa-sEVs influence human bone marrow-derived mesenchymal stem cells (hBMSCs), promoting their differentiation into myofibroblasts that secrete VEGF-A, hepatocyte growth factor (HGF), and matrix-modulating enzymes. These altered MSCs enhance angiogenic properties, leading to increased tumor growth and invasiveness in a three-dimensional model [64]. Considering the essential role of angiogenesis in tumor metastasis, targeting angiogenic sEVs, CAFs, and the ECM holds promise as a potential approach to halt tumor dissemination.

### Proliferation and metastasis

sEVs enhance intercellular communication by transferring RNA and proteins, which are vital in regulating PCa oncogenic processes, from cellular proliferation and metastasis (Fig. 1; Table 1).

#### RNA delivery

sEVs provide critical insights into the transcriptome of PCa cells, particularly in relation to RNA splicing. RNA-Seq on sEVs from CRPC plasma samples has identified mRNA isoforms linked to docetaxel resistance and disease progression [65] TUBB3 mRNA is elevated in plasma sEVs from mCRPC patients, correlating with shorter PSA progression-free survival (PSA-PFS) [66], while RNF157 mRNA in PC3-derived sEVs accelerates PCa growth by promoting macrophage M2 polarization [67]. sEVs can potentially alter the transcriptomic profile of recipient cells through transporting spliced variants, potentially disrupting the PCa TME, contributing to increased malignancy and recurrence. Notably, tumor-derived sEVs often carry miRNAs associated with oncogenic pathways, such as miR-375 [68, 69] and miR-18a-5p [70], which activate the Wnt/β-catenin pathway, promoting metastasis, proliferation and osteoblastic activity. CAFs transfer sEVs containing miR-1290 to PCa cells, promoting proliferation and metastasis by inhibiting the GSK3β/β-catenin pathway [71]. miRNAs, powerful regulators of gene expression, are transported via sEVs, amplifying their impact on recipient cells and potentially promoting tumorigenic signals or tumor suppressive activities. sEV-circRNA can remotely affect the miRNA environment and the transcriptional framework of target cells, potentially amplifying tumorigenic signals. sEVs transport circRNAs like circKDM4A [72] and circ-DHPS [73], which act as miRNA sponges, modulating key pathways to enhance proliferation, migration, and invasion. sEV-circ0081234 enhances the migration, invasion, and epithelial-mesenchymal transition (EMT) of PCa cells by modulating the miR-1/MAP3K1 axis [74]. Interestingly, sEVs-derived circRNAs not only act as miRNA sponges, regulating gene expression in recipient cells, but also enhance intercellular communication, influencing the behavior and functionality of recipient cells. CircTFDP2 correlates with Gleason score, metastasis status, and T- stage in PCa patients, promoting proliferation and metastasis [75]. Despite lacking protein-coding capacity, long non-coding RNAs (lncRNAs) offer potential therapeutic targets for PCa. Analysis of a lncRNA expression array in four mPCa cell lines revealed that sEVs are enriched with lncRNAs, particularly those containing seed regions for miRNAs such as the let-7 family, and miR-17, miR-18a, miR-20a, miR-93, and miR-106b [76]. The abundance of miRNA and RBP sites in sEV-lncRNAs significantly impacts PCa progression and metastasis. LncRNAs such as HOXD-AS1 [77], NEAT1 [78], and lncAY927529 [79] in sEVs regulate critical pathways, influencing metastasis, bone microenvironment modulation, and osteoinductive differentiation. These findings demonstrate that sEV-mediated transfer of specific mRNA, miRNA, circRNA and lncRNA in PCa progression play role by regulating critical signaling pathways. sEV-RNAs can indicate PCa malignancy and provide targets for metastasis treatment, with potential for discovering more RNAs in the future.

#### Protein delivery and PTM

As well as RNA, the multifunctional role of sEVs is also attributed to their abundant protein content. PCa-sEVs are powerful carriers of mRNA and proteins that can interfere with the tumor and the TME [80]. These vesicles transport bioactive enzymes and molecules, such as metalloproteinases, including membrane type 1 MMP (MT1-MMP, MMP14), a potent enzyme crucial for degrading the ECM, maintaining tissue balance, and facilitating cell invasion [81]. Under hypoxia, PC3-derived sEVs enhance MMP2, MMP9, fibronectin, and collagen activity, promoting pre-metastatic niche (PMN) and PCa metastasis [82]. Additionally, serum sEVs from PCa patients increase the release of extracellular MMP2, MMP9, and gamma-glutamyltransferase in various cell lines [83]. This intricate degradation of the ECM creates pathways for cancer cell migration, facilitating metastasis. Proteomic analyses have identified key sEV proteins, such as LRG1 [84] and ITGA2 [85], linked to angiogenesis and EMT, respectively. The presence of Cav-1 in tumor-derived sEVs also acts as a potent driver, inducing CSC phenotypes and EMT in PCa [86]. Furthermore, RelB was found to significantly enhance PCa cell aggressiveness via regulating sEV-ICAM1 [87, 88]. 

Apart from the proteins carried by sEVs, PTMs such as acetylation, glycosylation, palmitoylation, and SUMOylation can impact not only the function of these proteins but also their interaction with target cells. Acetylation of histone H3 at the CD274 promoter enhances sEV-PD-L1 secretion, driving immune evasion and PCa progression [89]. Glycosylation plays an essential role in both the biosynthesis and functional dynamics of sEVs [90], enhancing cargo delivery, protecting vesicles from enzymatic degradation, and prolonging their stability. Cavin-1 modulates sialic acid glycosylation in PC3-EVs, reducing their uptake by target cells and attenuating osteoclastogenic and osteoblastic activities [21]. A glycosylation motif improves the stability and expression of targeting peptide-Lamp2b fusion proteins in both cells and sEVs [91]. In PCa, the ratio of vesicle-associated PSA extraction is correlated with biantennary core-fucosylation. Variations in this ratio are linked to changes in N-glycoforms, highlighting its potential diagnostic significance [92]. Prostate-specific membrane antigen (PSMA), accumulates in sEVs, displaying increased glycosylation and partial proteolysis compared to cellular PSMA [93]. These variations highlight the potential use of N-glycosylation patterns as cancer biomarkers [94]. In-depth research on sEV glycosylation elucidates mechanisms of tumorigenesis and progression, offering new targets for tumor diagnosis and therapy. Palmitoylation, a well-known lipid modification, plays a crucial role in determining protein targeting and function within sEVs. Research has demonstrated that palmitoylation is essential for Dsg2 to regulate the sub-cellular localization of lipid raft and endosomal proteins involved in sEV biogenesis [95]. In cancer-initiating cells, Claudin7 (Cld7) is incorporated into tumor sEVs exclusively in its palmitoylated form, promoting tumor spread and metastasis [96]. Additionally, in PCa, specific palmitoyl-proteins such as STEAP1, STEAP2, and ABCC4 are predominantly found in sEV populations, they could promote cancer progression and sEV-mediated intercellular communication. Inhibiting palmitoylation in producing cells reduces the localization of these proteins in EVs, suggesting a pivotal role of palmitoylation in sorting EV-bound secretomes and offering potential pathways for selectively detecting disease biomarkers [97]. SUMOylation, a PTM of growing interest, has been observed to affect protein stability and functionality. Dysregulation of endogenous hexokinase 2 (HK2) SUMOylation may contribute to PCa cell proliferation and oncogenesis [98]. Similarly, Nucleus accumbens-associated 1 (NAC1) has been identified as a potential small SUMO substrate in PCa cells, with its multi-SUMOylation being crucial for PCa cell proliferation [99]. Heterogeneous nuclear ribonucleoprotein A2B1 (hnRNPA2B1) selectively binds to specific sEV-miRNAs by recognizing specific motifs and influences their encapsulation into sEVs. SUMOylated sEV-hnRNPA2B1enhances its ability to bind miR-198 [100]. These findings present opportunities for inovative targeted therapies that either disrupt or harness SUMOylation or other PTMs pathways in PCa. Novel regulatory mechanisms involving PTMs offer comprehensive insights into the roles of sEVs in tumorigenesis, metastasis, and the TME by regulating cell signaling pathways, modifying interaction with the ECM, and promoting PCa cell proliferation and invasion.

**Table 1:** PCa-sEV contents and functions

Content	Method	Source	Mechanism	Function	Reference
mRNA UBB3-201, CFL1-201, MIR222HG	RNA-Seq	PC3 cells & CRPC patients	Inflammation, apoptosis, lipid metabolism pathways	Promote docetaxel resistance	[65]
mRNA RNF157	NA	PC3 cells	HDAC1 ubiquitination, degradation, macrophage M2 polarization	Accelerate tumor growth	[67]
miR-222-3p	Small RNA transcriptomes	AIPC cells	MIDN/mTOR pathway	Promote CRPC progression	[101]
miR-375	RNA-seq, TCGA	Advanced PCa with bone metastasis, localized PCa & PCa cells	Wnt, PTPN4/STAT3 pathway	Promote osteoblastic metastasis, facilitate enzalutamide resistance	[68, 69]
miR-18a-5p	NA	C4-2B cells	Hist1h2bc/ Ctnnb1/Wnt/β-catenin pathway	Promote the differentiation of pre-osteoblasts towards osteoblasts and bone metastasis	[70]
miR-378a-3p	miRNA deep sequencing, miRNA-chip array	Benign prostatic hyperplasia (BPH), non-bone metastatic PCa, bone-metastatic PCa & PC3 cells	Dyrk1a/Nfatc1/Angptl2 axis	Enhance PCa proliferation and metastasis	[102, 103]
miR-92a-1-5p	Small-RNA sequencing	Osteoblastic, osteolytic, mixed PCa cells	COL1A1	Disrupt bone homeostasis, and promote tumor bone metastasis	[43]
miR-1290	RNA-seq	CAFs	GSK3β/β-catenin pathway	Promote PCa proliferation and metastasis	[71]
circKDM4A	NA	PCa patients	miR-338-3p/CUL4B axis	Promote PCa proliferation, migration, invasion tumorigenesis, and inhibit cell apoptosis	[72]
circ_0081234	circRNA microarray	MDA-PCa-2b cells	miR-1/MAP3K1 axis	Induce PCa migration, invasion and EMT	[74]
circ_SLC19A1	NA	22RV1 cells	miR-497/SEPT2/ ERK1/2 axis	Promote PCa growth and invasion	[104]
circ-DHPS	circBase, starBase v2.0, TargetScan	C4-2, PC3 cells	miR-214-3p/CCL5 axis	Promote PCa bone metastasis	[73]
circTFDP2	circRNA array	C4-2B, 22RV1 cells	PARP1/DNA damage axis	Promote PCa proliferation and metastasis	[75]
26 lncRNAs (ENST00000501280, uc010bys.1, uc001qgn.1 etc.)	Human 8 × 60 K LncRNA expression array	VCaP, LNCaP, DU145, PC3 cells	Harbor miRNA seed regions and RBP binding sites (ELAVL1 and RBMX)	Carcinogenesis and PCa diagnosis	[76]
lncRNA HOXD-AS1	Microarray analysis	LNCaP-AI, LNCaP-Bic cells & mPCa patients	WDR5, miR-361-5p/FOXM1 pathway	Promote PCa cell proliferation, migration, motility, metastasis and CRPC chemo-resistance	[77, 105]
lncRNA MIR222HG	RNA-seq	CRPC patients	Inflammation and apoptosis regulation Signals	Promote Docetaxel resistance	[65]
lincROR	RNA-seq	PC3 cells	MYH9/β-catenin/HIF1α regulatory axis	Promote DTX-resistant and tumor growth	[106, 107]
lncAY927529	Human 8 × 60 K LncRNA expression array	VCaP, LNCaP, DU145, PC3 cells & PCa patients	CXCL14/ p-ERK/ERK pathway	Promote cell proliferation, invasion and inhibite cell apoptosis, activate autophagy of bone marrow mesenchymal stem cells	[76, 79]
lncRNA NEAT1	Gene expression microarray dataset of mPCa (GSE38241), evsrbase	C4-2B cells & normal tissues, mPCa tissues	miR-205‐5p/RUNX2, SFPQ/PTBP2/ RUNX2 pathways	Promote hBMSC osteogenic differentiation	[78]
Protein ADAM7, AGRN, APP, et al.	LC/MS/MS	PC3 cells & PCa patients	MMP activation, ECM degradation, CD11b + cells accumulation	Remodel distant PMN, facilitate PCa cell invasion and metastasis	[82, 83]
Protein LRG1	Integrated proteomics and metabolomics	LNCaP cells & tumor-free controls (TFC), PCa, CRPC patients	NA	Facilitate the distant metastasis of advanced PCa	[84]
Protein ITGA2	NA	PCa patients & PC-3, DU145, LNCaP, CWR-R1 cells	ERK1/2/c-Myc axis	Increase PCa cell proliferation, migration, invasion, and reduced cell adhesion	[85]
Protein ICAM1	LC-MS analysis, UALCAN, GeneMANIA, tissue microarray analysis	DU145 cells	RelB-exo-ICAM1 axis	Increase DU145 aggressiveness and promote PCa progression	[88]
Protein PD-L1	NA	DU145 cells	p300/CBP/PD-L1 axis	Inhibit T cell function and induce resistance to anti-PD-L1 therapy	[89]

### Immune suppression and evasion

Tumor survival and progression rely on evading the host’s immune system [108], which is a complex mechanism. Tumor-derived sEVs have variable effects on immune cell activation, differentiation, and function, thus modulating the immune response to the tumor. For instance, PCa-sEVs expressing Fas ligand can induce apoptosis in CD8 + T cells [109]. PCa-sEVs also significantly influencing the activity of natural killer (NK) cells in the TME. PCa-sEVs containing NK cell protein group 2D (NKG2D) ligands decrease NKG2D levels on NK and CD8 + T cells, imparing their cytotoxicity thereby facilitating tumor immune evasion [110]. Additionally, a separate study demonstrated that levels of circulating EVs increase post- prostatectomy, enhancing NK cell activity by decreasing levels of NK cell protein group 2 A (NKG2A) and increasing NKG2D ligands [111]. Interestingly, heat-stressed tumor-derived sEVs (HS-TEXs) can exert anti-tumor effects by converting immunosuppressive regulatory T cells (Tregs) into pro-inflammatory Th17 cells via IL-6 signaling [112]. This indicates that the targeted use sEVs could potentially eliminate tumors. Nevertheless, it is important to acknowledge that the majority of tumor-derived sEVs primarily promote tumor progression. The uptake of sEV-PD-L1 via sEVs by tumor cells protects against CD8 + T cell attacks, suggesting that inhibiting sEV-mediated PD-L1 distribution may enhance the effectiveness of anti-PD-L1 therapy in PCa [113]. Moreover, sEV-IL-8 from PCa cells promotes immune evasion by disrupting CD8 + T cell glucolipid metabolism. It activates PPARα, reducing glucose utilization by downregulating GLUT1 and HK2, while increasing fatty acid breakdown via upregulation of CPT1A and ACOX1 [106]. PCa-sEVs also inhibit tumor antigen presentation by inducing CD73 expression on dendritic cells (DC), subsequently leading to immunosuppression [114]. Additionally, MHC class I-related chain molecules A and B (MICA/B), secreted via sEVs from PC3 cells, contributes to tumor immune evasion [115]. Collectively, PCa-sEVs inhibit immune cell function, consequently resulting in immune evasion. By regulating the influence of sEVs on CD8 + T cells, NK cells, etc., and elucidating sEV-mediated immune evasion mechanisms (such as the transfer of PD-L1 and NKG2D ligands), more potent immunotherapeutic approaches for PCa could be devised.

### Drug resistance

Therapeutic resistance, especially in PCa, remains a significant challenge in oncology. Resistance is frequently mediated through cellular and molecular adaptations. The involvement of sEVs in facilitating this resistance is becoming more acknowledged [116]. For example, Enzalutamide-resistant PCa cells release up to four times as many sEVs as sensitive cells. The heightened release of sEVs and the enhanced survival of Enz-resistant PCa cells might be linked to syntaxin 6. Administering GW4869 and dimethyl amiloride, which inhibit sEV production, significantly decreased the viability of these resistant cells [117]. Similarly, paclitaxel-resistant cells (PC3 and DU145) treated with the sEV GW4869 displayed a dose-dependent reduction in cell viability [118]. These findings suggest that blocking sEV release can sensitize resistant PCa cells to chemotherapy. sEVs act as biological messengers, carrying molecules, especially miRNAs, that enhance the defensive abilities of tumor cells [119]. These miRNAs, delivered via sEVs, can suppress the expression of drug targets or modify signaling pathways, reducing the efficacy of therapeutic agents. 29 altered miRNAs in sEVs from paclitaxel-resistant PCa cells (PC3 and DU145) likely regulate the genes AR, PTEN, and TCF4 in chemoresistant cells, compared to the miRNA profile of parent cells [120]. Additionally, sEV-circ-XIAP has been implicated in docetaxel resistance PCa by modulating the miR-1182/TPD52 axis [121]. sEVs derived from PSC-27 cells, enriched in miR-27a, were found to enhance chemoresistance by downregulating P53 gene expression [122]. Moreover, sEVs from PCa-associated fibroblasts carrying miR-423-5p increase resistance to taxane by inhibiting GREM2 via the TGF-β pathway. Notably, targeting the TGF-β pathway or inhibiting miR-423-5p can partially reverse this resistance, enhancing PCa cell susceptibility to chemotherapy, as demonstrated in vivo [123]. AR-V7, an active variant of the AR lacking a ligand-binding domain (LBD), is associated with resistance to specific hormonal therapies for PCa, including enzalutamide and abiraterone. Analysis of AR-V7 in plasma-derived sEVs from 36 mCRPC patients initiating treatment with these drugs revealed that detectable levels of AR-V7 significantly correlated with reduced overall survival (OS). Therefore, sEV-AR-V7 holds a promise as a biomarker for drug resistance [124, 125]. Another study on mCRPC patients found a correlation between sEV-TUBB3 mRNA expression levels and decreased response to abiraterone treatment [66]. PCa-sEVs enhance drug resistance in PCa by transporting diverse molecules. Investigating the specific mechanisms of sEVs in drug resistance and inhibiting their biogenesis or targeting key molecules has significant clinical implications for therapeutic applications.

## Non-tumor derived sEVs

**Fig. 2:**
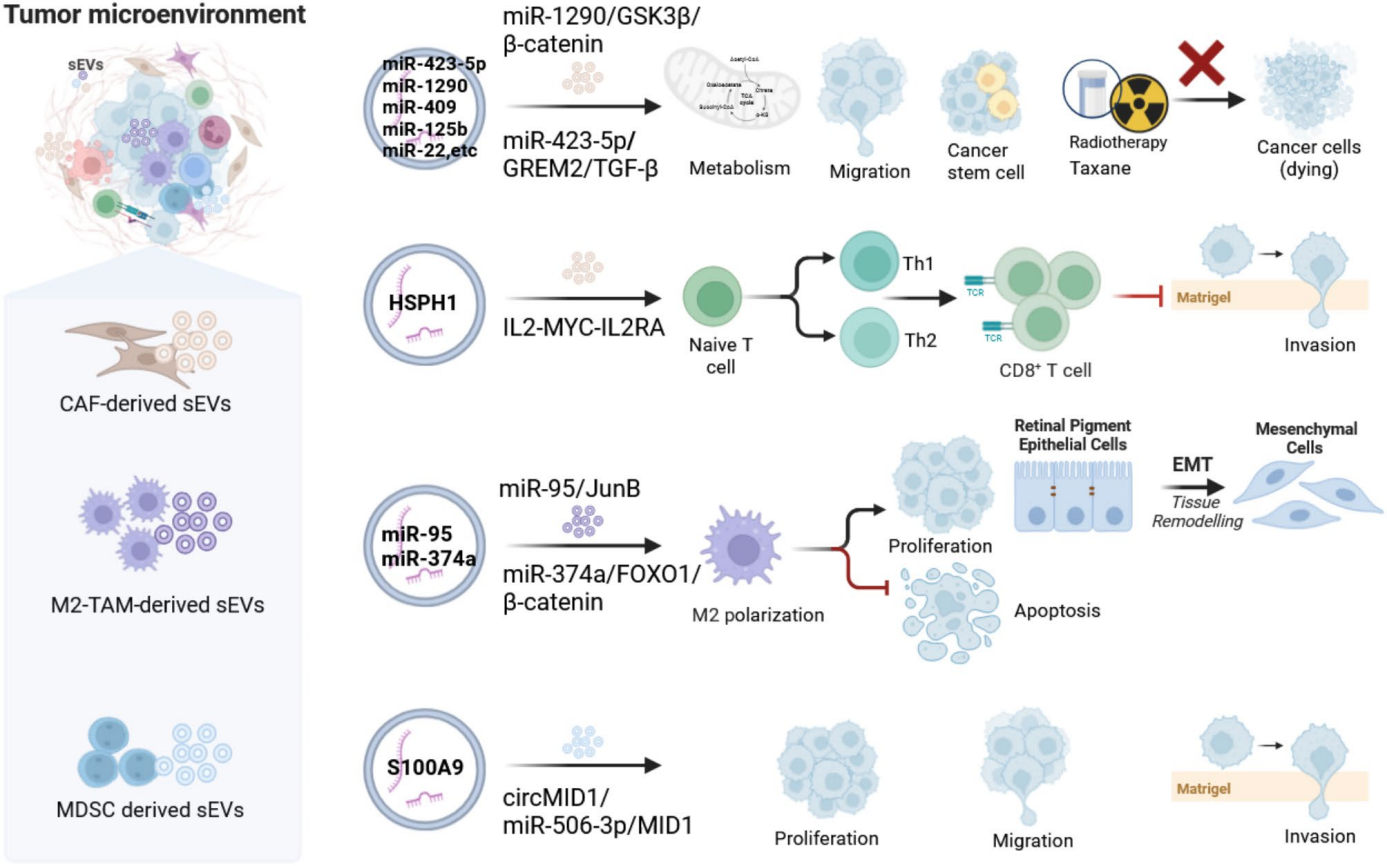
Non-tumor derived sEVs regulate PCa progression in the TME. In the TME, CAF-derived sEVs promote the malignant progression of PCa cells by carrying miRNAs but can also carry HSPH1, which inhibits invasion. M2-TAM-derived sEVs and MDSC-derived sEVs carry miRNAs and S100A9, facilitating the malignant progression of PCa

Actually, non-tumor derived sEVs are just as crucial as PCa-sEVs in remodeling TME homeostasis. Originally derived primarily from stromal and immune cells, non-tumor derived sEVs play a significant role in dynamic interactions within the TME through specific mechanisms (Fig. 2).

### Stromal-cell derived sEVs

The stroma serves as the structural support for biological tissues, with stromal cells, particularly fibroblasts and endothelial cells, playing significant roles in maintaining TME balance. CAFs have a notable influence on the TME in different types of cancers such as breast, colorectal, and PCa, through ECM remodeling and sEV secretion [126–128]. Importantly, CAF-sEVs also contribute to the establishment of an immunosuppressive TME, enabling cancer cells to evade immune surveillance [121, 129]. In PCa, sEVs from stromal cells surrounding the prostate exhibit pro-tumor properties [123]. Initial studies on PCa histology have emphasized the importance of mRNA signatures from stromal-derived EVs. A study identified 19 unique transcripts by comparing vesicles from normal and disease-associated stromal cultures. Combining specific mRNAs with PSA using machine learning (ML) has improved assay accuracy in predicting disease progression [130]. Recent research has shed light on the complex communication network within the TME, where CAFs, normal fibroblasts, and cancer cells release miRNA-loaded-sEVs [131]. Specifically, EVs from fibroblasts carry unique miRNAs that modulate critical signaling pathways essential for cancer progression [132]. The exact mechanisms through which CAFs influence PCa tumorigenesis are still largely unknown. While early-stage PCa typical shows a positive response to androgen deprivation therapy (ADT), the development of castration resistance and metastasis is almost inevitable [133]. Studies have indicated that stromal cells predominantly enhance the radioresistance of PCa cells through sEV-mediated delivery of IL-8 [41]. Additionally, PCa-CAF-derived EVs enriched with miR-423-5p promote taxane resistance in PCa cells by modulating GREM2 through the TGF-β pathway [123]. The role of sEV-miRNAs in cancer progression is becoming clearer. Downregulation of sEV-miR-146a-5p from CAFs has been found to enhance the EMT process and accelerate cancer spread via the EGFR/ERK pathway [134]. Similarly, stromal fibroblasts mainly promote tumorigenesis, EMT, and stemness in epithelial cancer cells through miR-409 [135]. Further studies have identified miR-1290 from CAF-derived sEVs as a significant promoter of PCa cell growth and metastasis via the GSK3β/β-catenin signaling pathway [71]. CAF-derived sEVs not only promote PCa malignancy through enhancing drug resistance and pathways related to EMT, but are also abundant in several miRNAs, including miR-22, let-7a, and miR-125b. These miRNAs are recognized for their ability to inhibit mitochondrial oxidative phosphorylation and modulate metabolic pathways in PCa cells [136]. Stromal-cell-derived sEVs, particularly from CAFs, have been demonstrated to promote tumorigenesis, metastasis, and drug resistance through transferring mRNA, miRNA, and modulating metabolic pathways. This accumulating evidence highlights the pivotal role of CAF-derived miRNAs in the complex dynamics of PCa progression.

### Immune-cell derived sEVs

The tumor-immune microenvironment (TIME) in PCa typically exhibits immunosuppressive characteristics, mainly characterized by TAMs, Tregs, and myeloid-derived suppressor cells (MDSCs) [137]. RNA-seq and digital pathology investigations have identified that infiltrating Tregs and macrophages in the PCa TIME are linked to unfavorable prognosis [138]. Immune cell-derived sEVs play a role in regulating immune responses by transporting cytokines, antibodies, immune-modulating factors and sEVs. In PCa, both adaptive and innate immune cells play crucial role in the disease initiation, progression, metastasis, and treatment [139]. TAMs, for example, promote PCa growth by transferring miR-95 via sEVs, facilitating cell proliferation, invasion, and the EMT process via miR-95/JunB axis [42]. Similarly, miR-374a within the sEVs secreted by M2 macrophages promotes the EMT in PCa cells [140]. ​​ Furthermore, MDSCs-sEVs promote CRPC progression through the circMID1/miR-506-3p/MID1 axis, including increased cell proliferation, migration, and invasion [141]. Although research on the impacts of immune cell-derived sEVs in PCa is limited, emerging findings suggest novel therapeutic approaches could be unveiled. Genetic engineering and chemical modification of sEVs improve tumor-specific targeting through presentation of targeted ligands or bioactive proteins like cytokines and antibodies on their surface [142, 143]. This approach holds the potential to revive exhausted CD8 + T cells and bolster immune responses against tumors [144]. sEVs derived from PCa cells efficiently anchored IFN-γ fusion proteins on their surface, leading to an increased proportions of CD4+, CD8+, and IFN-γ + CD8 + T cells, as well as M1 macrophages, suggesting a strengthened immune response [145]. Akkermansia muciniphila (Akk), a Gram-negative anaerobic bacterium, effectively activates CD8 + T lymphocytes and shifts macrophages towards an M1-like phenotype, stimulating antitumor immunity in a streamlined manner [146]. Utilizing sEVs derived from immune cells within the complex TIME of PCa shows promise for developing targeted therapies, including boosting immune cell function and enchancing tumor-specific targeting.

## Cancer diagnosis and prognosis

**Fig. 3:**
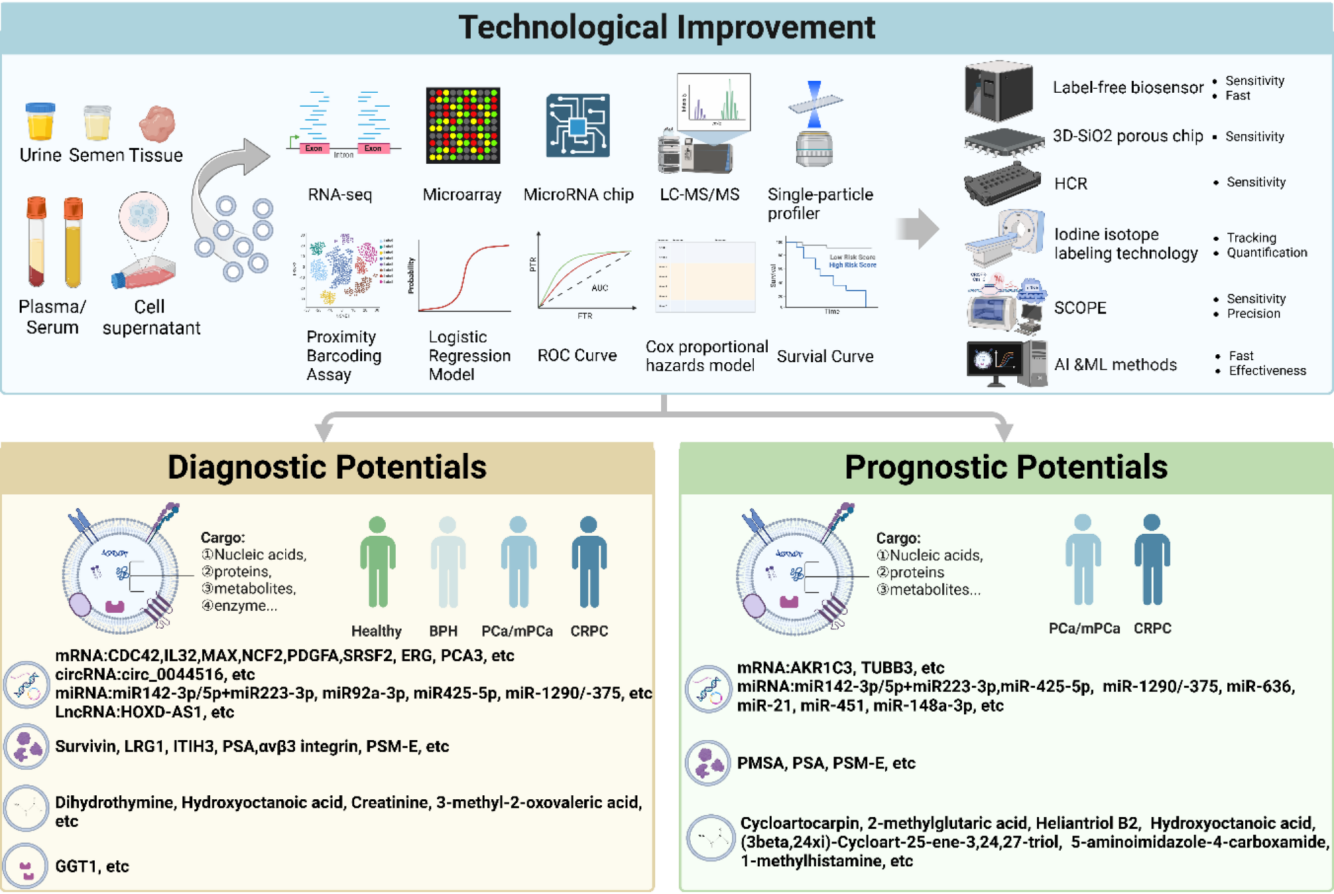
Diagnostic and Prognostic Potentials of sEVs in PCa: Advances in Detection Methods and Biomarker Identification. sEVs derived from various body fluids and tissues can be identified and analyzed using techniques such as RT-PCR, ddPCR, RNA-seq, Microarray, MicroRNA chips, SPP, and PBA. Candidate biomarkers are screened through methods like ROC analysis and survival curves. Advanced technologies, including HCR, SCOPE, and approaches integrating AI and ML methods, are employed to enhance detection sensitivity and specificity. A wide range of nucleic acids, proteins, metabolites, and enzymes can be utilized to diagnose and differentiate PCa and CRPC, while also providing prognostic insights for PCa and CRPC management

The diagnosis of PCa continues to pose challenges due to the invasive nature and associated risks of conventional tissue biopsies. PSA screening has improved PCa detection rates, but it also leads to high rates of false positives and false negatives. Consequently, liquid biopsies have emerged as a revolutionary approach in oncology, offering a less invasive option. sEVs, containing a wealth of molecular information (Table 2; Fig. 3), are increasingly recongnized as a valuable tool in liquid biopsies for both diagnosing PCa and predicting its prognosis.

### Early screening and diagnosis

Early detection of PCa is vital for optimal therapeutic outcomes. sEV biomarkers exhibit higher specificity and sensitivity compared to traditional PSA testing [147]. For example, sEV proteins, such as LAMB1 in blood and Histone H4 in urine, demonstrated greatly efficacy in diagnosing PCa compared to serum PSA [18]. Moreover, Survivin [148] and GGT1 [149] showed higher serum sEV activity in PCa patients compared to BPH patients, suggesting that serum sEV Survivin/GGT activity may serve as a potential biomarker for PCa. Likewise, urinary sEV-associated PSM-E is significantly upregulated in PCa patients, correlating with high Gleason scores and advanced TNM stages, and demonstrates excellent diagnostic performance (AUC = 0.8904) [150]. A study has revealed unique characteristics of sEV-mRNA compared to tissue mRNAs. A logistic regression model incorporating an sEV-mRNA signature (CDC42, IL32, MAX, NCF2, PDGFA, SRSF2) attained an AUC of 0.948, successfully discriminiating between PCa patients and healthy individuals [151]. Similarly, circ_0044516 was significantly elevated in sEVs derived from PCa patients and associated cell lines, indicating its potential as a marker for PCa [47]. sEV miRNAs serve as novel and reliable biomarkers for PCa diagnosis and treatment. Among patients with chronic prostatitis/chronic pelvic pain syndrome (CP/CPPS) type IIIb, miRNAs in sEVs from blood and post-prostatic-massage urine showed elevated levels of eight PCa-specific miRNAs, including hsa-miR-501, hsa-miR-20a, and hsa-miR-106. These miRNAs target genes are significantly associated with oncogenic processes, indicating their potential in early PCa diagnosis [152]. Unique miRNA profiles in seminal fluid sEVs can differentiate PCa from samples obtained from healthy individuals. Models that integrate PSA with miR-142-3p, miR-142-5p, and miR-223-3p achieved an AUC of 0.821 for PCa detection, while combinations incorporating miR-342-3p and miR-374b-5p distinguished higher-grade tumors with an AUC of 0.891, thereby improving diagnostic precision for PCa [153]. Emerging evidence suggests that miRNAs enclosed within sEVs facilitate molecular communication between tumors and metastatic sites. Specific miRNAs are linked to CRPC and PCa metastasis, with miR-423-3p indicating CRPC [154], miR-425-5p tied to tumor stages [155], and the ratios of miR-150-5p [156], miR-194-5p/miR-16-5p [157] associated with metastasis and disease severity. Importantly, miR-125a-3p, miR-330-3p, miR-339-5p, miR-613 and miR-92a-3p have been identified as potential biomarkers for PCa bone metastasis [158, 159]. In addition to miRNA derived from sEVs in plasma and tissues, urinary sEV miRNA profiles from 149 PCa patients identified miR-21, miR-451, miR-636, alongside preoperative PSA levels, showing promise as noninvasive diagnostic markers for PCa and its metastatic potential [160, 161]. A urinary sEV gene expression assay in 499 participants effectively distinguished high-grade from low-grade PCa and benign conditions by targeting ERG, PCA3, and SPDEF [162, 163]. Integrated proteomics and metabolomics analysis of plasma-derived sEVs from TFC, PCa, and CRPC patients revealed that apolipoprotein E levels were 1.7 times higher in PCa samples compared to TFC, with elevated levels of LRG1 and ITIH3 in CRPC compared to PCa. Additionally, differential metabolites were identified as diagnostic markers for distinguishing these conditions (Fig. 3) [84]. The contents of sEVs often frequently mirror the tumor’s state. Continuous monitoring of these changes enables clinicians to obtain immediate understanding of PCa dynamics and adapt treatments as needed, potentially serving as an early warning system. It is necessary to prospectively evaluate diagnostic assays to ensure efficacy and clinical adoption.

### Prognostic assessment

Biochemical recurrence (BCR) can occur after the surgery in PCa. Predicting the trajectory of PCa, from indolent to aggressive states, is crucial for personalized treatment planning. Using digital droplet polymerase chain reaction (ddPCR), positive AKR1C3-sEVs expression in blood samples correlated with decreased survival rates, indicating an unfavorable prognosis for both OS and PFS under first-line abiraterone use (ABI-PFS) [164]. In mCRPC patients administrated with abiraterone, higher levels of sEV-TUBB3 mRNA were associated with shorter PSA-PFS, with negative TUBB3 having a mean of 11.0 months and strong TUBB3 expression having a mean of 3.6 months (*P* = 0.005) [66]. High expression of has-miR-148a-3p in sEV miRNAs from Russian CRPC patients is associated with increased risk of CRPC progression (HR = 2.05, *P* = 0.005) [165]. RNA sequencing on a screening cohort of 23 CRPC patients identified miR-1290/-375 as candidates associated with OS. Incorporating these miRNAs into models based on clinical prognostic factors significantly improved predictive performance, Increasing the time-dependent AUC from 0.66 to 0.73 for predicting OS in CRPC patients [166]. Moreover, certain biomolecules encapsulated within sEVs can indicate tumor response to specific treatments, offering insights into PCa patient prognosis. The presence of αvβ3 integrin on sEVs, associated with aggressive cancer traits across various cancer types, suggests its potential as a non-invasive marker for monitoring PCa progression [167, 168]. Different contents transported by sEVs can be used to assess the risk of tumor recurrence and response to treatment. sEVs have emerged as a highly promising non-invasive biomarker with significant potential in the prognosis and ongoing monitoring of PCa.

**Table 2:** Potential diagnostic and prognostic marker for PCa

Type	Component	Source	Cohort Design	Level	Significance	Clinical Application	Reference
Protein	Survivin	Plasma	39 PCa vs. 8 recurrent vs. 20 BPH vs. 16 healthy controls	↑	Gleason 6 or Gleason 9 scores > BPH and healthy controls & chemotherapy relapse patients overexpressed	Early Diagnosis & chemotherapy resistance	[148]
	Apolipoprotein E, LRG1, ITIH3	Plasma	TFC vs. PCa vs. CRPC	↑	PCa > TFC (Apolipoprotein E, ROC value 0.74) & CRPC > PCa (LRG1, ITIH3, ROC values 0.84, 0.85, respectively)	Diagnosis of PCa from TFC & prediction of CRPC from PCa	[84]
	αvβ3 integrin & CD9	Plasma	PCa vs. age-matched individuals without cancer	↑	PCa > age-matched healthy individuals	Diagnosis for PCa	[167]
	LAMB1	Plasma	15 controls vs. 30 localised primary PCa vs. 15 mPCa	↑	mPCa > controls (healthy and BPH participants) (*P* < 0.0001) & localised primary PCa (*P* < 0.0001)	PCa diagnosis and risk stratification	[18]
	Histone H4	Urine	15 low-risk PCa vs. 15 high-risk PCa	↑	High-risk > low‐risk PCa (*P* < 0.0001)	PCa diagnosis and risk stratification	[18]
	PSM-E	Serum, urine	45 controls vs. 48 PCa	↑	AUC of 0.8904 for PCa vs. controls	Early diagnosis and prognosis for PCa	
Enzyme	GGT1	Plasma	31 PCa vs. 8 BPH	↑	PCa > BPH	Diagnosis for PCa & differentiating PCa from BPH	[149]
mRNA	CDC42, IL32, MAX, NCF2, PDGFA, SRSF2	Plasma	141 PCa vs. 170 BPH vs. 30 healthy controls	↑	AUC of 0.948 for PCa vs. healthy controls	Diagnosis for distinguishing PCa from BPH and healthy controls	[151]
	ERG, PCA3	Urine	255 training cohort, 519 validation cohort	↑	sEVs + standard of care (SOC) (PSA level, age, race, family history) (AUC 0.77), SOC alone (AUC 0.66) (*P* < 0.001)	Diagnosis for high-grade PCa	[163]
	AKR1C3	Plasma	62 mCRPC	↑	ABI-PFS: 3.9 vs. 10.1 months, *P* < 0.001 & OS: 16.2 vs. 32.5 months, *P* < 0.001	Prognosis for mCRPC	[164]
	TUBB3	Plasma	52 mCRPC using abiraterone as first-line therapy	↑	Positive TUBB3 expression, PSA-PFS (11.0 vs. 7.9 months; *p* = 0.014). Strongly TUBB3 (> 20 copies/20 µl), PSA-PFS (11.0 vs. 8.3 vs. 3.6 months; *p* = 0.005).	Prognosis under abiraterone treatment	[66]
circRNA	circ_0044516	Plasma	50 PCa vs. normal controls	↑	PCa > normal controls	Diagnosis for PCa	[47]
miRNA	miR-150-5p	Plasma	31 PCa vs.12 healthy donors	↑	PCa > healthy controls (AUC:0.89)	Diagnosis for high-risk PCa	[156]
	miR-23b-3p	Plasma	31 PCa vs. 12 healthy donors	↓	Gleason 5 > Gleason 7	Diagnosis for advanced PCa	[156]
	miR-181a-5p	Serum	BPH vs. non-bone metastatic PCa or bone-metastatic PCa	↑	PCa diagnosis (AUC: 0.856) & bone metastatic PCa diagnosis (AUC:0.738)	Diagnosis for bone- metastatic PCa	[102]
	miR-142-3p, miR-142-5p, miR-223-3p	Semen	31 PCa vs. BPH vs. healthy controls	↑	PSA + miR-142-3p + miR-142-5p + miR-223-3p, (AUC: 0.821) & high-grade tumors (AUC: 0.891)	Diagnosis and prognosis for PCa	[153]
	miR-425-5p	Cell	mPCa cells and sEVs vs. nontumor samples	↑	Pathologic T stage, pathologic N stage, and residual tumor Samples overexpressed	Prognosis for bone metastasis in PCa	[155]
	miR-1290/-375	Plasma	123 CRPC patients	↑	AUC: 0.73	Prognosis for CRPC patients	[166]
	miR-636, miR-21, miR-451	Urine	112 PCa (75 localized, 37 metastatic) & 37 PCa (27 localized, 10 metastatic)	↑↓	AUC (0.925, *n* = 112) & AUC (0.896, *n* = 37) for predicting metastasis (miR-636, miR-21, miR-451, PSA)	Prognosis for metastasis in PCa	[160]
	miR-148a-3p	Plasma	11 mCRPC during therapy (docetaxel/abiraterone)	↓	CRPC progression (HR = 2.05, *P* < 0.005)	Prognosis in PCa therapy resistance	[165]
LncRNA	HOXD-AS1	Serum	38 localized vs. 92 metastatic (before initial treatment)	↑	Metastatic > localized PCa patients & shorter PSA recurrence-free survival (PRFS, *P* = 0.006, HR = 2.05) and PFS (*P* = 0.02, HR = 2.27)​	Prognosis for PCa	[77]

### Diagnostic and therapeutic technologies

PSA testing has limitations in early PCa detection, leading to overtreatment or missed diagnoses due to its low sensitivity, particularly in the gray zone. Nanovesicles called PSA-sEVs, which are released in response to microenvironmental acidity and express both PSA and sEV marker CD81, offer a potential non-invasive method for early PCa detection [45, 169]. A recent study presented a label-free biosensor using plasmonic metasurfaces and antibodies against PSA and CD63 to identify serum sEVs. This portable system detects serum PSA and sEVs in 20 min and outperforms the conventional PSA test with a sensitivity of 92.3% for early PCa [170]. The Proximity barcoding assay (PBA) identified specific protein combinations unique to Prostasomes, including CD166/CD63 and ADAM10/CD166. These markers distinguish Prostasomes from other sEV sources and enable differentiation between PCa patients and healthy individuals, highlighting PBA’s utility for precise, high-throughput analysis in heterogeneous samples [171]. The integration of nanoscale porous properties and multiple sEV-specific markers in a 3D-SiO2 porous chip significantly enhances biosensing sensitivity [172]. This increased precision strengthens the diagnostic potential of sEVs, facilitating early detection. Urinary sEV miRNAs have shown promise as biomarkers, but their low concentrations in clinical samples pose challenges. By employing a hydrogel-based hybridization chain reaction (HCR) for multiplex signal amplification, minute quantities of miRNAs, such as hsa-miR-6090 and hsa-miR-3665, can be detected from 600 µL of urine with up to 35-fold amplification. This method significantly improved detection limits [173]. The Self-amplified and CRISPR-aided Operation to Profile EVs (SCOPE) technology further advances this field by leveraging the CRISPR-Cas13 system, using crRNA to guide Cas13 in recognizing target RNA and amplifying signals. This enables highly sensitive EV mRNA detection with single-nucleotide resolution and demonstrates broad applicability in precision medicine, including lung and colorectal cancer, showcasing its broad applicability in precision medicine [174]. Additionally, advancements in other research areas include the development of a streamlined iodine isotope labeling technique, enabling non-invasive tracking and quantification of tumor lesions and EVs in animal models [175]. Surface-Enhanced Raman Spectroscopy (SERS) has recently garnered considerable attention as a highly sensitive and label-free method for sEV analysis for cancer diagnosis [176]. Kim WH et al. [177] revealed that a 3D SERS sensor can accurately discriminate PCa patients from healthy controls with a diagnostic accuracy of 0.93, based on varying levels of urinary sEV miRNAs. Another research developed an Au-coated TiO2 macroporous inverse opal (MIO) structure, designed with an engineered slow light effect, leading to exceptional SERS performance. Validation indicated that the intensity of the 1087 cm-1 SERS peak from sEVs in the plasma of cancer patients (prostate, lung, liver, and colon) is at least double that of healthy individuals. This approach offers notable advantages, such as being noninvasive and time-efficient, compared to currently utilized clinical tumor liquid biopsy techniques [178]. Compared to CTCs, which are primarily found in blood and exist in extremely low quantities in peripheral circulation [179], sEVs are more stable, easily isolated from various biofluids, and ideal for non-invasive PCa diagnostics. Unlike ctDNA, which is limited in early-stage cancer, sEVs carry a broader range of biomolecules, reflecting real-time tumor dynamics [180]. Additionally, sEVs can be used for targeted drug delivery. Incorporating artificial intelligence (AI), ML—a subset of AI, utilizes algorithms to analyze data, derive insights from it, and create models to assist in predictions and decision-making. For example, a specific combination of five mRNAs (CAV1, THBS1, CTGF, TIMP2, and AKT1) that accurately distinguished between high and low Gleason scores, outperforming the accuracy of PSA [130]. sEVs offer promising platform for liquid biopsies can be easily detected in body fluids such as blood and urine [181]. Elevated levels of specific molecules within sEVs, such as miRNAs or proteins, often indicate the presence of PCa cells. Detection of these molecules in circulating sEVs can serve as diagnostic indicators, providing a comprehensive molecular fingerprint that helps clinicians accurately determine tumor type and stage. Compared to traditional PSA tests, sEV-based biomarkers may offer higher specificity and sensitivity (Fig. 3). They not only act as early indicators but also provide valuable insights into tumor classification, staging, and treatment response.

## Therapeutic prospects

sEVs are increasingly recognized as innovative therapeutic platforms in the treatment of PCa. Engineered sEVs have been modified to carry specific drugs, genes, or proteins to achieve targeted therapy, enhancing their specificity and efficacy. These engineered sEVs are being explored across several cutting-edge therapeutic modalities, including precision drug delivery, immunotherapy, and stem cell-derived interventions.

### Drug delivery therapy

sEVs, with their biocompatibility and ability to traverse biological barriers, have gained considerable interest as potential carriers for drug delivery. Especially, sEVs derived from the body’s own cells exhibit minimal immunogenicity, making them excellent candidates for drug delivery [182]. sEVs derived from LNCaP and PC3 cells efficiently deliver paclitaxel back to their cells of origin, enhancing its uptake and cytotoxic effects via the endocytic pathway [22]. Furthermore, sEVs containing the tumor suppressor Maspin further highlight the diverse therapeutic potential of these vesicles [183]. Targeting and are crucial factor in therapeutic interventions, and the inherent targeting capabilities of sEVs offer a promising platform for precision drug delivery. Engineered sEVs can target tumor-specific markers like PSMA in PCa. Anti-PSMA sEV mimetics (EMs), created by genetically modifying U937 cells and extruding them, have demonstrated their potential for PCa treatment in both in vitro and in vivo studies [184]. The vesicles can be engineered to carry a variety of therapeutics, including chemotherapeutics, small molecules, and nucleic acid, providing a multipronged approach to targeting tumor cells. Aptamer-modified sEVs have been developed to deliver siRNA, effectively silencing SIRT6 and inhibiting tumor growth and metastasis in xenograft mouse models [23]. Spherical nucleic acids (SNAs), innovative nanomaterials with a gold core and oligonucleotide shell, can be synthesized to counteract miR-21. By loading SNAs into sEVs using an sEV-endosomal pathway, anti-miR21 exo-SNAs are created. These exo-SNAs have shown to reduce miR-21 expression by 50% in PC3 cells, demonstrating the potential of engineered synthetic sEVs as delivery vehicles for targeted therapies [185]. Combining sEVs with polyethylenimines (PEIs) enhances siRNA or antimiR delivery. sEV-modified PEI complexes targeting miR-155 or miR-1246 have showed increased efficacy in reducing tumor growth in PC3 PCa mouse models [24]. Another exciting development is a urine-derived sEV nanocarrier, Exo-PMA/Fe-HSA@DOX, designed for homologous targeting of PCa by combining low-dose chemotherapy with photodynamic therapy [186]. This multifunctional sEV-based nanocarrier specifically targets tumors by delivering various types of therapeutics, offering a novel strategy for PCa treatment.

### Immunotherapy for PCa

sEVs can be employed to modulate the immune system, enhancing its ability to recognize and attack prostate tumors [106]. Treatment with sEV biogenesis inhibitor GW4869 significantly inhibits the release of PCa cell-derived sEVs, impedes macrophage M2 polarization, and suppresses PCa metastasis [187]. Wang D et al. [188] demonstrated that an ultrasound-based drug delivery strategy using sEVs encapsulated with sonosensitizers Chlorin e6 and immune adjuvant R848 can enhance anti-tumor immunity. Ultrasonic irradiation not only enhanced R848-mediated DC maturation but also shifted macrophages from an immunosuppressive M2-like phenotype to an anti-tumor M1-like phenotype in a synergistic manner. A combination therapy using TGFβRI kinase inhibitor SD-208 and TLR-7/8 agonist R848 was investigated using serum-derived sEVs (EXOs) as versatile carriers. SD-208/EXOs and R848/EXOs reduced the migration of B16F10 and PC3 cells and triggered the release of proinflammatory cytokines from stimulated macrophages and DCs [189]. Furthermore, engineered sEVs can deliver checkpoint inhibitors, facilitating robust tumor targeting by blocking inhibitory pathways. This approach is further supported by a dual-mode liquid biopsy strategy that combines peptide engineering with nanoscale assessment, as described in recent research [190]. Previous research has demonstrated that enhancing the immunogenicity of sEVs can be achieved by targeting the localization of antigens. BN Immuno Therapeutics is developing MVA-BN-PRO, a next-generation immunotherapeutic that encodes two tumor-associated antigens, PSA and prostatic acid phosphatase (PAP), higher protection rates or prolonged survival [191, 192]. Administering viruses encoding sEV-directed PSA or PAP to mice resulted in a higher frequency of PSA- or PAP-specific T cells compared to wild-type transgenes [192]. Tumor-derived sEVs carrying tumor-associated antigens hold promise for immunotherapy. Researchers have developed an sEV vaccine using a protein-anchoring method with sEVs derived from PCa cells. The IFN-γ-sEV vaccine, using PCa-sEVs, enhanced M1 macrophage activity and antibody production, leading to clearance of PCa-sEVs, reduction in Treg levels, suppression of tumor-promoting expressions, and ultimately, inhibition of tumor growth and improved survival in mice with PCa [145]. The US FDA has approved Provenge(^®^) (sipuleucel-T), a novel PCa vaccine that utilizes antigen-presenting cell technology with DC cells [193]. Combined treatment with R848 and another immune adjuvant or checkpoint inhibitor, such as a PD-1/PD-L1 inhibitor, using sEVs as promising carriers, can be a convincing strategy to circumvent tumor growth in vivo, and immunotherapy holds practical significance in inhibiting PCa progression.

### Stem-cell derived sEVs in PCa therapy

**Fig. 4:**
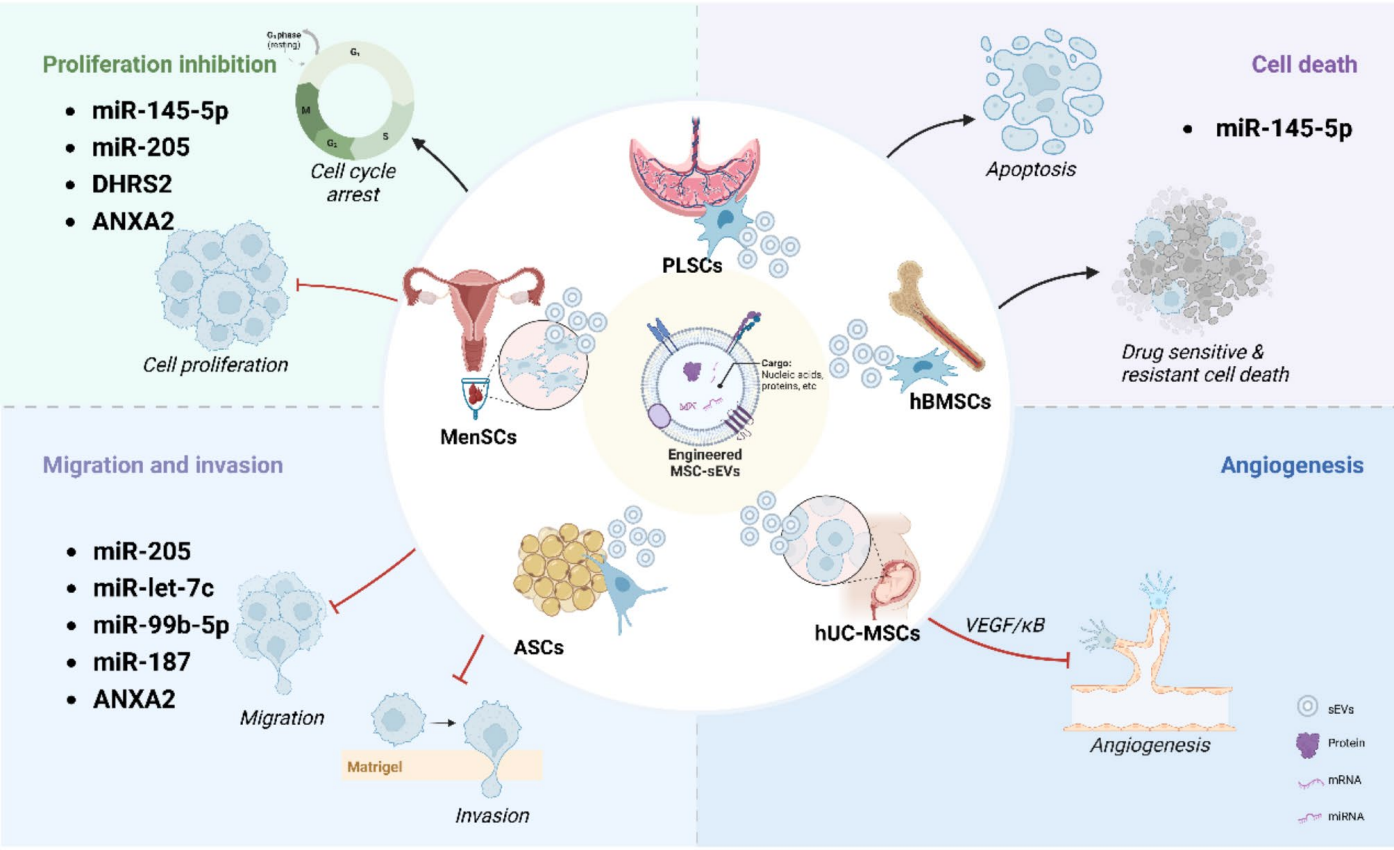
Therapeutic Potential of Stem-cell Derived sEVs in PCa Treatment. sEVs derived from hBMSCs, huc-MSCs, ACSs, MenSCs, and PLSCs, either naturally or engineered to carry miRNAs, proteins, and other bioactive molecules, can inhibit PCa proliferation, angiogenesis, invasion, metastasis, and drug resistance, while promoting apoptosis. (All figures were created with BioRender.com)

Stem cells have regenerative capabilities and release sEVs that reflect this remarkable attribute [194]. These sEVs contain growth factors, cytokines, and specific miRNAs that promote tissue regeneration, which is crucial after injury or therapeutic interventions. It is intriguing to speculate that stem-cell sEVs also direct cell fate within the TME [195], the molecular cargo carried by these vesicles could impact cell differentiation, facilitating the transformation into less malignant forms. Several studies have suggested that stem-cell-derived sEVs can counteract PCa malignant transformation (Fig. 4). For example, sEVs from placental stem cells (PLSCs) specifically suppress aggressive PCa cells, significant reducing the survival of both sensitive and resistant PCa cell lines without affecting healthy cells [196]. Certain stem cell derived sEVs have shown promising effects in modulating angiogenesis in the TME [197]. MSC-secreted sEVs have been found to suppress in vitro angiogenesis by modulating the mTOR/HIF-1α/VEGF signaling pathway [198]. NF-κB signaling has been identified as a crucial mediator of MSC-sEVs induced angiogenesis in endothelial cells [199]. Menstrual stem cells (MenSCs) secreted sEVs reduce VEGF secretion and NF-κB activity, suppressing the secretion of pro-angiogenic factors by PC3 cells in a reactive oxygen species (ROS)-dependent manner [200]. sEV therapy derived from adipose-stromal cells (ASCs), a type of MSC, exhibits dose- and time-dependent antitumor effects on PCa lines, inducing cell cycle arrest and apoptosis. The therapeutic potential of ASC-sEVs is further supported by their efficacy in bladder and renal cancer cells [25]. Research has found that ASC-derived sEVs carrying miR-145-5p inhibit PCa growth and promote apoptosis by targeting BclxL [201, 202]. Similarly, hBMSC-derived sEVs carrying miR-205 inhibit PCa cell proliferation, invasion, and migration while promoting apoptosis. The downregulation of RHPN2 by sEV-miR-205 further validates its in vivo efficacy [203, 204]. Researchers are exploring miRNA-based antitumor strategies using engineered MSC-derived sEVs loaded with RNA. For instance, exogenous miR-let-7c packaged into hBMSC-derived sEVs significantly reduces cell proliferation and migration in CRPC-like PC3 and CWR22RV1 cells [205]. Moreover, hBMSC-derived sEVs modified with miR-99b-5p mimics [206] and miR-187 [207] collectively inhibit PCa progression by targeting the miR-99b-5p/IGF1R axis and the miR-187/CD276/JAK3-STAT3-Slug signaling pathway, respectively, thereby suppressing cancer cell viability, proliferation, invasion, and migration. Additionally, DHRS2-modified sEVs derived from human umbilical cord mesenchymal stem cells (hUC-MSCs) suppress PCa cell growth and increase apoptosis, demonstrating their potential as a treatment option [208]. Studies indicate that specific sEVs from stem cells can enhance the response of PCa cells to chemotherapy, potentially improving treatment outcomes. Nanovesicles derived from induced pluripotent stem cell-derived mesenchymal stem cells (iPSC-MSCs) show great potential in delivering the chemotherapy drug docetaxel, increasing its effectiveness against resistant PCa cells [209], and this approach holds promise for treating advanced PCa. Within TME, one critical aspect of MSCs’ therapeutic benefits is their ability to modulate the immune homeostasis [210, 211]. These vesicles could restore the immune system’s capacity to recognize and attack PCa cells. For instance, ANXA2-enriched sEVs produced by culturing and engineering BMSCs from BALB/c nude mice with ANXA2-loaded lentiviral plasmids effectively suppressed the growth, invasion, and migration of PCa cells and reduced tumor growth in the mice by targeting M2 macrophages [212]. Collectively, stem cell-derived sEVs offer promising therapeutic strategies for PCa. They enhance the precision of drug delivery, effectively transport therapeutic RNAs to silence specific oncogenes, adjust the immune response to reestablish tumor immunity homeostasis in the TME, and hinder tumor progression, thereby improving treatment outcomes.

## Conclusions and challenges

The exploration of sEVs in PCa presents numerous opportunities along with significant challenges. The heterogeneity and multifunctional attributes of sEVs highlight their essential role in modulating the homeostasis of the TME in PCa.

sEVs hold considerable promise as biomarkers for the early detection and prognosis of PCa. The rich and diverse molecular cargo within sEVs, encompassing specific proteins, miRNAs, and circRNAs, offers more accurate and sensitive diagnostic means compared to conventional methods such as PSA testing. sEVs can be engineered as efficient drug delivery vehicles, facilitating the targeted delivery of chemotherapeutic agents, small molecules, and nucleic acids. This targeted approach has the potential to enhance treatment efficacy and minimize adverse effects. Building on this foundation, even smaller nanovesicles or particles, such as supermeres, have been identified. Moreover, sEVs can be utilized in immunotherapy to regulate the immune system and augment its ability to target prostate tumors. They can carry immune-modulating factors and checkpoint inhibitors to strengthen anti-tumor immune responses. Stem cell-derived sEVs show potential in inhibiting cancer growth, regulating angiogenesis, and influencing the immune response within the TME, offering novel therapeutic avenues.

Over the years, there has been significant progress in isolating and evaluating sEVs, driving technological advancements. Nanoparticle tracking analysis and tunable resistive pulse sensing have significantly improved the accuracy and efficiency of sEV characterization and quantification. Proteomics, lipidomics, and RNA sequencing have allowed for a deeper understanding of the molecular composition of sEVs. Advanced imaging techniques, including cryo-electron microscopy, fluorescence labeling, micro imaging and microfluidic chip have been crucial in visualizing sEV ultrastructure [213]. Vortex-induced convection enhances sEV separation by accelerating particle transport to the liquid-liquid interface in systems like ATPS, overcoming diffusion limitations [214]. However, the heterogeneity of sEVs in aspects such as their size, shape, cargo, and functions pose substantial challenges in their characterization and understanding. This complexity makes the standardization of isolation and quantification techniques difficult, leading to inconsistent research outcomes and hindrances in clinical application. The rise of AI has enabled the identification of potential disease-specific biomarkers, enhancing the focus and efficiency of liquid biopsies [215, 216]. By applying ML algorithms along with techniques such as mass spectrometry and sequencing, sEV-derived data can be rapidly analyzed to uncover trends or patterns associated with target diseases, thereby improving the precision and effectiveness of sEV-based liquid biopsies. For example, an explainable AI-based screening system using urinary sEV biomarkers improved PI-RADS 3 diagnosis accuracy, achieving an AUC of 0.93 [217]. Single-particle profiler (SPP) is a high-throughput method for analyzing nanoscale particles, providing detailed data on payload distribution, encapsulation efficiency, and biophysical properties, with applications in LNPs, antibody-virus binding, and nanoparticle research [218]. 

The clinical application of sEVs requires standardization in isolation, characterization, and analysis. The International Society for Extracellular Vesicles (ISEV) has established the MISEV2023 guidelines, which provide recommendations on sample collection, isolation techniques (e.g., ultracentrifugation, size-exclusion chromatography), characterization methods (e.g., Atomic Force Microscopy (AFM), Dynamic Light Scattering (DLS), Electron Microscopy (EM), Nanoparticle Tracking Analysis (NTA), Transmission Electron Microscopy (TEM), and Western blot for sEV markers like CD9, CD63, CD81, TSG101, ALIX), and data reporting to ensure reproducibility., and data reporting to ensure reproducibility. Despite these advancements, the absence of a consensus on the optimal methods for isolating and quantifying sEVs impedes the reproducibility of research and the translation of laboratory findings into clinical practices [219]. Standardized protocols for different source materials and quality control measures for sEV functionality need to be established [220]. The precise mechanisms by which sEVs interact with the TME and contribute to PCa progression are not fully elucidated, emphasizing the significance of clarifying these processes for the development of effective therapeutic strategies. Ensuring the targeted delivery of therapeutic sEVs to the tumor site or their homing to the appropriate location remains a major obstacle, which is crucial for optimizing the therapeutic effect of sEV-based interventions. Translational barriers include the necessity for large-scale clinical cohort studies to validate the clinical utility of sEV applications, rigorous preclinical testing to understand the pharmacodynamics and potential toxicities. Overcoming these challenges will require interdisciplinary cooperation and technological advancements to fully exploit the potential of sEVs in the diagnosis and treatment of PCa.

## Data Availability

No datasets were generated or analysed during the current study.

## Abbreviations

ADT: Androgen deprivation therapy
AFM: Atomic force microscopy
AI: Artificial intelligence
ASC: Adipose-stromal cells
BCR: Biochemical recurrence
BPH: Benign prostatic hyperplasia
CAF: Cancer-associated fibroblasts
CAV1: Caveolin 1
CRPC: Castration-resistant prostate cancer
DC: Dendritic cells
ddPCR: Digital droplet polymerase chain reaction
DLS: Dynamic light scattering
ECM: Extracellular matrix
EM: Electron microscopy
EMT: Epithelial-mesenchymal transition
ESCRT: Endosomal sorting complex required for transport
hBMSCs: Human bone marrow-derived mesenchymal stem cells
HGF: Hepatocyte growth factor
HMEC1: Human microvascular endothelial cells
hMSCs: Human mesenchymal stem cells
HUVECs: Human umbilical vein endothelial cells
iPSC-MSCs: Induced pluripotent stem cell-derived mesenchymal stem cells
ITIH3: Inter-Alpha-trypsin inhibitor heavy chain H3
LBD: Ligand-binding domain
lncRNA: Long non-coding RNAs
LRG1: Leucine-rich alpha2-glycoprotein 1
MDSC: Myeloid-Derived suppressor cells
NF-κB: Nuclear factor kappa B
miRNA: MicroRNA
ML: Machine learning
MMP: Matrix metalloproteinase
MVBs: Multivesicular bodies
NKG2A: NK cell protein group 2 A
NKG2D: NK cell protein group 2D
NK: Natural killer
NTA: Nanoparticle tracking analysis
OS: Overall survival
PAP: Prostatic acid phosphatase
PBA: Proximity barcoding assay
PCa: Prostate cancer
PCa-sEVs: Prostate cancer-derived small extracellular vesicles
PEI: Polyethylenimines
PD-L1: Programmed death-ligand 1
PFS: Progression-free survival
PLSC: Placental stem cells
PMN: Pre-metastatic niches
PrSC: Prostate stromal cells
PSA: Prostate-specific antigen
PSMA: Prostate-specific membrane antigen
SERS: Surface-enhanced Raman spectroscopy
sEV: Small extracellular vesicles
SNA: Spherical nucleic acids
SOC: Standard of care
SPP: Single-particle profiler
TAM: Tumor-associated macrophages
TEM: Transmission electron microscopy
TFC: Tumor-free controls
TIME: Tumor-immune microenvironment
TME: Tumor microenvironment
VEGF: Vascular endothelial growth factor
